# Chitin Deacetylases: Structures, Specificities, and Biotech Applications

**DOI:** 10.3390/polym10040352

**Published:** 2018-03-22

**Authors:** Laia Grifoll-Romero, Sergi Pascual, Hugo Aragunde, Xevi Biarnés, Antoni Planas

**Affiliations:** Laboratory of Biochemistry, Institut Químic de Sarrià, Universitat Ramon Llull, 08017 Barcelona, Spain; laiagrifollr@iqs.edu (L.G.-R.); sergipascualt@iqs.edu (S.P.); hugoaragunde@gmail.com (H.A.); xavier.biarnes@iqs.edu (X.B.)

**Keywords:** chitin deacetylases, chitosan, chitooligosaccharides, carbohydrate esterases, structure, substrate specificity, deacetylation pattern

## Abstract

Depolymerization and de-*N*-acetylation of chitin by chitinases and deacetylases generates a series of derivatives including chitosans and chitooligosaccharides (COS), which are involved in molecular recognition events such as modulation of cell signaling and morphogenesis, immune responses, and host-pathogen interactions. Chitosans and COS are also attractive scaffolds for the development of bionanomaterials for drug/gene delivery and tissue engineering applications. Most of the biological activities associated with COS seem to be largely dependent not only on the degree of polymerization but also on the acetylation pattern, which defines the charge density and distribution of GlcNAc and GlcNH_2_ moieties in chitosans and COS. Chitin de-*N*-acetylases (CDAs) catalyze the hydrolysis of the acetamido group in GlcNAc residues of chitin, chitosan, and COS. The deacetylation patterns are diverse, some CDAs being specific for single positions, others showing multiple attack, processivity or random actions. This review summarizes the current knowledge on substrate specificity of bacterial and fungal CDAs, focusing on the structural and molecular aspects of their modes of action. Understanding the structural determinants of specificity will not only contribute to unravelling structure-function relationships, but also to use and engineer CDAs as biocatalysts for the production of tailor-made chitosans and COS for a growing number of applications.

## 1. Introduction

Chitin is a linear polysaccharide of β(1→4)-linked *N*-acetylglucosamine monomers. It was first isolated from fungi in 1811 [1] and its structure was determined in 1929 [2]. Chitin is a major structural component of the exoskeletons of arthropods (insects and crustaceans), of the endoskeletons of mollusks, and it is also found in the cell walls of fungi and diatoms [1,3,4]. It is one of the most abundant organic molecules after cellulose, and the most abundant natural amino polysaccharide. Chitin is present as ordered macrofibrils, mainly in two allomorphs: α-chitin, with antiparallel chains [5], is the most abundant and it is isolated from the exoskeleton of crustaceans, particularly from shrimps and crabs; and β-chitin, with parallel chains [6], is present in the cell walls of diatoms and in the skeletal structures of cephalopods, and commonly extracted from squid pens. β-Chitin is easily converted to α-chitin by alkaline treatment followed by flushing in water [5]. Chitin is also found as γ-chitin in fungi and yeast, which is a combination of the α and β allomorphs [7,8].

**Figure 1:**
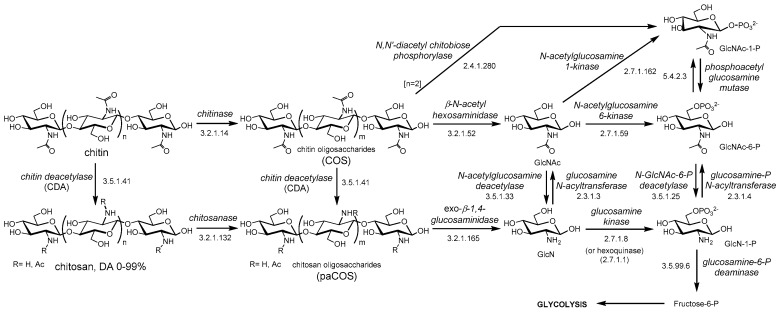
Chitin catabolism. GlcNAc: *N*-acetylglucosamine; GlcN: glucosamine; DA: degree of acetylation. COS: chitooligosaccharides (or chitin oligosaccharides), paCOS: partially acetylated chitooligosaccharides (or chitosan oligosaccharides).

Depolymerization (hydrolysis) of chitin by chitinases results in chitooligosaccharides (COS), and de-*N*-acetylation of chitin and COS yields chitosan and partially acetylated COS (paCOS) or fully deacetylated glucosamine oligomers (Figure 1). In nature, the deacetylation of chitin is almost never complete, and chitosan refers to a family of heteropolysaccharides composed of *N*-acetylglucosamine and glucosamine, characterized by their degree of polymerization (DP), degree of acetylation (DA), and pattern of acetylation (PA). Only some fungi of the *Zygomycota*, *Basidiomycota* and *Ascomycota* phyla have been reported to be capable of naturally producing chitosans [9]. The free amino groups of the deacetylated units in the polymer are protonated at slightly acidic pH, thus making chitosans the only known natural polycationic polysaccharides [1,3,9]. They interact with polyanionic biomolecules such as polyanionic phospholipidic membranes, glycosaminoglycans at cell surfaces, proteins, and nucleic acids. Depolymerization by hydrolysis of the β-1,4-linkages of chitin and chitosan yields their respective oligosaccharides (COS, paCOS) [10]. Whereas chitin and chitosans act as structural polymers, their oligomers are involved in molecular recognition events, such as cell signaling and morphogenesis, and act as immune response elicitors and host-pathogen mediators [11,12,13,14,15]. The catabolism of chitin and chitosan is summarized in Figure 1. Chitin and chitosan oligosaccharides are further degraded following different organism-dependent pathways to end up in the central energy metabolism.

The de-*N*-acetylation of chitin and chitooligosaccharides is catalyzed by chitin deacetylases (CDAs), which exhibit different substrate specificities leading to fully or partially deacetylated products with diverse degrees of acetylation (DA) and patterns of acetylation (PA). In addition to the role CDAs play in the biology of their natural organisms, there is a growing interest in the biochemical characterization of CDAs in order to use them as biocatalysts for the production of partially deacetylated chitooligosaccharides (paCOS) as bioactive molecules in different application fields, or to inhibit them since they are potential targets against pathogenic microorganisms. The aim of this review is to analyze the current knowledge on the biochemistry of chitin deacetylases with regard to their substrate specificity. Although a large number of enzymes have been experimentally identified as chitin deacetylases able to deacetylate chitin either in vivo or in vitro, few of them have been analyzed at the protein level. Here we will focus on those CDAs with characterized activity on chitooligosaccharides (COS) to address the issue of substrate specificity and the deacetylation pattern.

## 2. Chitin Deacetylases and the Carbohydrate Esterase Family 4 (CE4) 

Chitin deacetylases (CDAs, EC 3.5.1.41) and chitooligosaccharides deacetylases (CODs, EC 3.5.1.105) are classified in the carbohydrate esterase family 4 (CE4) in the CAZY database (Carbohydrate Active Enzymes, www.cazy.org) [16]. The CE4 family also contains peptidoglycan *N*-acetylglucosamine deacetylases (EC 3.5.1.104), peptidoglycan *N*-acetylmuramic acid deacetylases (EC 3.5.1.-), poly-β-1,6-*N*-acetylglucosamine deacetylase (EC 3.5.1.-), and some acetyl xylan esterases (EC 3.1.1.72) [17]. These enzymes share a conserved region known as the NodB homology domain due to its similarity to the NodB oligosaccharide deacetylase, one of the first CE4 enzymes to be characterized [18]. Most currently reported and characterized CDAs and CODs are CE4 enzymes, with the exception of diacetylchitobiose deacetylases (Dacs) from archaea and a COD from *Bacillus cereus* (*Bc*ZBP) that belong to the CE14 family [19,20]. Few other enzymes, such as insect CDAs and a COD from *E. coli* (ChbG) [21] are in the group of “non-classified” in the CAZY database since they do not share sequence similarities to the other CDA families.

The deacetylase activity from extracts of the fungus *Mucor rouxii* was the first active CDA identified and partially purified in the mid 1970s [22,23]. Later on, the NodB from a rhizobium species was the first biochemically characterized COD in 1993 [18]. Many other CDAs and CODs were later identified and purified from very diverse organisms, including archaea, marine bacteria, fungi, and insects [24]. These enzymes are diverse in their biochemical properties: molecular masses in the range from 12 to 150 kDa, acidic isoelectric points (pI from 2.7 to 4.8), optimum pH for activity from 4.5 to 12, and significant thermal stability, with optimum temperatures for activity in the range from 30 to 60 °C. Most CDAs are highly inactive on crystalline chitin due to the inaccessibility of the acetyl groups in the tightly packed chitin structure, and have a preference for soluble forms of chitins such as glycol-chitin or chitin oligomers, as well as partially deacetylated chitin (chitosans). It has recently been shown that CDA activity on crystalline chitin is greatly enhanced by oxidative cleavage of the surface polymer chains by lytic polysaccharide monooxygenases (LPMO) [25]. Some CDAs contain carbohydrate binding modules (CBM) fused to the catalytic domain that seem to enhance the deacetylase activity by increasing the accessibility of the substrate to the catalytic domain [26].

CDAs are localized in different cellular compartments, in the periplasm, in the cytosol, or secreted as extracellular enzymes. Periplasmic fungal CDAs are generally tightly coupled to a chitin synthase to rapidly deacetylate newly synthesized chitin before their maturation and crystallization. Extracellular fungal CDAs are secreted to alter the physicochemical properties of the cell wall, which results in protection against exogenous chitinases, or initiates sporulation or autolysis. In bacteria, CDAs are either intracellular, as those involved in Nod factors biosynthesis in *Rhizobium* species, or extracellular, as those involved in the catabolism of chitin in marine bacteria [24,27,28].

Some CE4 enzymes classified in a specific subfamily also show activity on typical substrates from other subfamilies. Peptidoglycan GlcNAc deacetylases, involved in the de-*N*-acetylation of the bacterial cell wall peptidoglycan with critical functions in the maturation and turnover of peptidoglycan and in bacterial pathogenicity, are also active on COS. Some CDAs have activity on acetylxylan, as well as some acetylxylan esterases are active on COS, which makes their classification doubtful in some cases.

## 3. Function and Specificity of CE4 Chitin Deacetylases

### 3.1. Deacetylation Patterns

Chitin deacetylases exhibit diverse deacetylation patterns, reflecting different substrate specificities and pattern recognition on their linear substrates. The mechanisms of action of enzymes that modify in-chain units on a linear polysaccharide are commonly classified as multiple-attack, multiple-chain, and single-chain mechanisms [29]. In the multiple-attack mechanism, binding of the enzyme to the polysaccharide chain is followed by a number of sequential deacetylations, after which the enzyme binds to another region of the polymeric chain. (i.e., *M. rouxii* [30,31]). On a polymeric substrate, this mechanism will result in a block-copolymer structure with blocks of GlcNH_2_ units within the GlcNAc chain. On COS, it will usually result in full deacetylation of the oligomer. In the multiple-chain mechanism, the enzyme forms an active enzyme-polymer complex and catalyzes the hydrolysis of only one acetyl group before it dissociates and forms a new active complex (i.e., *C. lindemuthianum* CDA [32,33]). It will result in a random distribution of the GlcNH_2_ and GlcNAc units along the polymeric chain or, in the case of COS substrates, it will render a number of partially deacetylated oligosaccharide intermediates ending in a specific deacetylation pattern or in full deacetylation, depending on the enzyme and the substrate. Finally, a single-chain mechanism includes processive enzymes in which a number of catalytic events occur on a single substrate molecule leading to sequential deacetylation. Some bacterial chitooligosaccharides deacetylases (CODs), which are specific for a single position leading to mono-deacetylated products (i.e., *Rhizobium* NodB or *Vibrio* CDA or COD) are also included in the last group.

A fundamental question is how these enzymes define their action pattern. This is relevant not only to understand their biological functions but also to use CDAs (native and engineered variants) as biocatalysts for the production of chitosans with non-random deacetylation patterns, and partially deacetylated COS with tailored patterns of acetylation (see Section 6). A structural model on the determinants of substrate specificity is currently emerging from studies on substrate specificity, determination of 3D structures of enzyme-substrate complexes, and multiple sequence alignments. Many CDAs, particularly from fungal origin, have been identified as involved in chitin deacetylation in vivo, but only few of them have been characterized with regard to substrate specificity and mode of action. To the best of our knowledge, Table 1 compiles the CDA enzymes in family CE4 that have been biochemically characterized and have reported activity on COS substrates, some of them with solved three-dimensional structure by X-ray crystallography. Relevant information on their biological function and substrate specificity is summarized below.

CDAs participate in diverse biological processes, which include cell wall morphogenesis and host-pathogen interaction in fungi, generation of signaling molecules in bacteria, and participation in the catabolism of chitin as carbon, nitrogen, and energy sources in marine bacteria and fungi. CDAs were thought to be restricted to fungi and bacteria until a first report in 1986 on their presence in arthropods [34]. CDAs seem to be widely present in insects, in cuticles and the perithrophic midgut matrix, but little is known on the function and properties of insect CDAs [35,36], and they are not included here because scarce information on substrate specificities has been reported.

### 3.2. Fungal CDAs

Fungal CDAs are involved in fungal nutrition, morphogenesis and development [27,29], participating in cell wall formation and integrity [37], in spore formation [38], germling adhesion [39], fungal autolysis [40], and in defense mechanisms for host infection [41].

Fungi that have chitosan (in addition to chitin) as a structural component of the cell wall, secrete CDAs to the periplasmic space that contribute to chitosan biosynthesis from nascent chitin synthesized by chitin synthases. It occurs during exclusive periods corresponding to their particular biological role in the cell cycle of the fungal species: during cell wall formation (i.e., *M*. *rouxi* [42] and *A. coerulea* [43]), during sporulation (i.e., *S*. *cerevisiae* [38,44]), or during vegetative growth (i.e., *C*. *neoformas* [37]).

Pathogenic fungi secrete CDAs during fungal hyphae penetration to evade plant defense mechanisms and gain access to host tissues. Plants secrete chitinases to break the fungal cell-wall chitin down to chitooligosaccharides (COS), and the released COS are recognized by plant chitin-specific receptors, triggering resistance responses [41]. COS elicitation of resistance mechanisms involve activation of host defense genes [45,46]. There is cumulative evidence that fungi evade plant defense mechanisms by partially deacetylating either their exposed cell wall chitin or the chitooligosaccharides produced by the action of plant chitinases. In both cases, the resulting partially deacetylated oligomers are not well recognized by the specific plant receptors reducing or preventing the elicitation of the defense responses [41,47,48,49].

Filamentous fungi undergo autolysis by self-digestion of aged hyphal cultures due to carbon starvation [40,50]. During this event there is an increased presence of hydrolytic enzymes, especially those involved in cell wall degradation, and CDAs are secreted to the extracellular medium to deacetylate the chitin oligomers produced by chitinases (i.e., *A*. *nidulans* [51,52]).

In general, fungal CDAs deacetylate soluble forms of chitin such as glycol-chitin and chitosans of variable DA, but they are inactive, or show low activity, towards insoluble chitins such as crystalline α- and β-chitin and colloidal chitin. Pretreatment of chitin to make surface fibrils more accessible may result in increased deacetylase activity. It has been recently shown that the activity of an *A. nidulans* CDA on crystalline chitin was enhanced by a lytic polysaccharide monooxygenase (LPMO) that increases substrate accessibility by oxidative cleavage of the chitin chains [25]. Some CDAs also appear to be active on acetylxylan (i.e., *An*CDA), but any of them act on peptidoglycans, typical substrates of other CE4 family members. In addition to polymeric substrate, not many CDA have been analyzed with COS as substrates (Table 1). The analysis of the products from enzymatic deacetylation with regards to the extent and pattern of deacetylated provides information about the specificity and mode of action of these enzymes. The first seven entries in Table 1 correspond to CDAs for which the deacetylation pattern on COS has been reported, whereas the rest of the entries are CDAs active on COS but, to the best of our knowledge, the structure of the deacetylated products has not been analyzed.

*Mucor rouxii* (*Amylomyces rouxii*) (*Mr*CDA). The dimorphic fungus *M. rouxii* has a cell wall mainly composed of chitin, chitosan, and mucoric acid. While chitin accounts for 10% of the total dry weight of the cell wall, chitosan reaches 30% [77]. The *M. rouxii* CDA enzyme was initially found in the cytosol [23], but it is also secreted into the periplasm where it participates in a tandem synthetic mechanism that involves a chitin synthase and a chitin deacetylase working consecutively and synchronously for synthesis and deposition of chitosan polymers at the outer membrane [78]. Decoupling this mechanism prevents the formation of chitosan [42,79]. *M. rouxii*, like other fungi, has been identified as a suitable microorganism for chitosan production by means of biofermentation processes [80,81]. *Mr*CDA is a monomeric high-mannose-type glycosylated protein with an apparent molecular mass of 75–80 kDa [82]. Kinetic studies indicate that the preferred catalytic metal is Zn^2+^ like many other CDAs. In terms of activity, its optimal pH and temperature values are between 4.5–5.5 and 50 °C, respectively [23,33]. *Mr*CDA deacetylates chitinous polymers such as glycol-chitin, colloidal chitin, chitosan, and chitin, but also deacetylates acetylxylan [17,23]. On chitooligosaccharides, triacetylchitotriose is the smallest substrate and the activity increases with the degree of polymerization (DP) [23,31,42,78]. The enzyme follows a multiple-attack mechanism [30] but the resulting pattern of acetylation (PA) depends on the DP of the substrate: whereas DP3, DP6 and DP7 substrates are not fully deacetylated, leaving the reducing GlcNAc unmodified [D_n_−_1_A], DP4 and DP5 substrates are fully deacetylated [D_n_]. In all cases, deacetylation starts at the non-reducing end residue and then proceeds to the neighboring monomer towards the reducing end [31].

*Colletotrichum lindemuthianum* (*Cl*CDA). The deuteromycete *C. lindemuthianum* is a plant pathogen that causes anthracnose, a disease which affects economically important crop species [83]. *Cl*CDA is a heavily glycosylated secreted enzyme allegedly playing a role in the host-pathogen interaction, deacetylating the chitin oligomers resulting from the activity of plant chitinases on the fungal cell wall [83,84]. Less likely is its function in deacetylating the fungal cell wall chitin to evade degradation by plant chitinases, since no chitosan has been observed in the cell wall ultra-structure [85]. Since its discovery in the 1980s it has been purified from its natural host [84,86] as well as expressed in several eukaryotic and bacterial hosts such as *Pichia pastoris* [87,88] and *E. coli* [89,90]. The enzyme has a preference for Co^2+^ and Zn^2+^ as the catalytic metal cation and its activity is substantially inhibited by Cu^2+^ or Ni^2+^, but not inhibited by EDTA or acetate [53,86]. It is a quite thermostable enzyme with an optimum temperature of 60 °C, and a pH optimum of 8.0. *Cl*CDA is active on both chitin polymers (glycol-chitin) and COS. It fully deacetylates COS with a DP equal to or greater than 3, while it only deacetylates the non-reducing GlcNAc of diacetylchitobiose [32,91]. *Cl*CDA acts by a multiple-chain mechanism following a pathway in which the first residue to be deacetylated is the second from the reducing end [32,33]. The initial mono-deacetylation reaction shows no dependency of k_cat_ on DP and a decrease of K_M_ with increasing DP [33,53]. However, kinetics of fully deacetylated products formation show an increase in k_cat_ and reduction in K_M_ that correlate with the increase of DP [86]. It has been reported that this enzyme is reversible, as it is also able to catalyze the acetylation of chitosan oligomers [92,93,94].

*Aspergillus nidulans* (*An*CDA). During cell autolysis, *An*CDA is secreted into the extracellular medium to deacetylate the chitin oligomers produced by chitinases [40,50,52,95]. The enzyme has been purified from A. nidulans cultures as a glycosylated enzyme [51]. The recombinant protein has been expressed in E. coli and purified by refolding from inclusion bodies [96] and recently it has been obtained in soluble form [25]. Like ClCDA, *An*CDA is a thermostable protein with an optimal temperature of 50 °C and retaining 68% activity after 1 h at 80 °C. Its optimum pH is 7–8 [51,96]. The enzyme is active on soluble chitins (CM-chitin, glycol-chitin), colloidal chitin, chitosan, acetylxylan, and acetylated glucuronoxylan, but not on peptidoglycan [25,51]. *An*CDA is active on COS with a DP from 2 to 6 [25]. The enzyme catalyzes mono-deacetylation of (GlcNAc)_2_ and it is inactive on GlcNAc monosaccharide. Longer substrates than DP2 are fully deacetylated. However, the deacetylation rate exhibits a counter-intuitive relationship with the DP of the substrate: odd-numbered COS (DP5, DP3) have higher apparent rate constants than even-numbered oligomers (DP4, DP2). For the DP6 substrate, time-course monitoring of products formation reveals that the first deacetylation event occurs at random positions except for the reducing end, which reacts much slower to yield the fully deacetylated end product [D_n_].

*Podospora anserina* (*Pa*CDA). The filamentous ascomycete *Podospora anserina* lives as a saprophyte on herbivore dung [97]. It has a limited lifespan and it is a model organism in cell aging studies [98]. *Pa*CDA was identified in a search for CDAs containing chitin binding domains. The enzyme has been recombinantly expressed in *Hansenula polymorpha* as a full length protein composed of the CE4 domain flanked by two CBM18 domains [26]. The low activity of the enzyme on colloidal chitin is significantly reduced by deletion of the CBM domains, which supports the hypothesis that the presence of the CBMs helps the enzyme to act on insoluble substrates. *Pa*CDA is active on soluble glycol-chitin, chitosans with a high DA, and COS, with optimum pH and temperature values of 8.0 and 55 °C, respectively. It fully deacetylates COS with a DP ≥ 2 and follows a multiple-chain mechanism. With the DP3 substrate, the first deacetylation event has a clear preference for the reducing end, but all possible isomers are found for both mono- and di-deacetylated intermediate products. With DP4 and DP5 substrates, the residue next to the reducing end is preferentially deacetylated first, with the second deacetylation occurring mainly next to the existing GlcNH_2_ unit on either side. Deacetylation is faster for longer substrates, with deacetylation of the reducing end occurring as a late event [26].

*Puccinia graminis f.* sp. *Tritici* (*Pgt*CDA). The biotrophic basidiomycete *Puccinia graminis f.* sp. *Tritici* is the causative agent of the stem rust [99]. The appearance of resistant races of *P. graminis* affecting wheat cultivars has been recognized as a serious threat to food security [100,101], boosting the interest in understanding the virulence and defense mechanism of this fungal pathogen. Rust fungi promote the formation of complex structures in order to invade the plant cells but at the same time they must prevent the triggering of immune responses [102]. A main transition during infection is from the ectophytically growing appressorium to the endophytically growing substomatal vesicle; while the former exposes chitin on its surface, the latter exposes chitosan [47,103]. *Pgt*CDA may not only participate in the chitin to chitosan transition, making the cell wall less susceptible to host chitinases [104], but also could deacetylate the chitooligosaccharide products, reducing its elicitor properties [105]. *Pgt*CDA has been recombinantly expressed in *E. coli* as a fusion protein with the maltose binding protein (MBP) [54]. Its optimal pH for activity is between 8 and 9 and its optimal temperature is 50 °C. It is not active on insoluble polymers such as α- or β-chitin, but efficiently deacetylates colloidal chitin, glycol-chitin and chitosans, on which activity increases with the degree of acetylation. With COS substrates, the minimal substrate is tetraacetylchitotetraose (DP4). The structure of the products from enzymatic deacetylation of DP4 to DP6 substrates reveals that the enzyme acts by a multiple-chain mechanism and specifically deacetylates all but the last two GlcNAc units on the non-reducing end [AA(D)_n_−_2_] [54].

*Pestalotiopsis* sp. (*Pes*CDA). The endophytic fungus *Pestalotiopsis* sp. lives inside the tissues of its plant hosts in tropical areas [106]. To successfully survive in their hosts, endophytes also need to avoid being detected by the plant immune system. A secreted *Pestalotiopsis* CDA has been identified when chitosan was present in the culture medium [49]. The recombinantly expressed *Pes*CDA is active on colloidal chitin as substrate, chitosans with a DA of 10–60% (higher activity with a higher DA), and COS, but inactive on crystalline α- or β-chitin. When analyzing the activity on COS, tetraacetylchitotetraose is the minimal substrate. With a DP5 substrate, the optimum pH and temperature values are 8.0 and 55 °C, respectively. Through a multiple-chain mechanism, the enzyme deacetylates all residues of the substrates except the reducing end and the last two GlcNAc residues from the non-reducing end, with a pattern of deacetylation [AA(D)_n_−_3_A] [49]. The chitosan oligomers obtained from deacetylation of a DP6 substrate by *Pes*CDA have shown that, as opposed to the fully acetylated oligomer, they are no longer elicitors of the plant immune system in rice cells [49].

*Pochonia chlamydosporia* (*Pc*CDA). The ascomycete *Pochonia chlamydosporia* infects females and eggs of cyst or root-knot nematodes. It is used as a biocontrol agent against a number of plant parasitic nematodes in food-security crops [107,108,109]. *P. chlamydosporia* expresses chitosanases and chitin deacetylases during egg infection. Since chitosan is associated with the sites of fungal penetration, it has been suggested that secreted CDAs are involved in nematode infection [110]. A *Pc*CDA has been recently characterized [55]. The full-length protein contains the CE4 catalytic domain flanked by two CBM18 chitin binding domains. The recombinantly expressed *Pc*CDA catalytic domain deacetylates COS with a DP ≥ 4, with preference for longer substrates. It starts deacetylating the penultimate residue from the non-reducing end and continues deacetylating the next residue towards the reducing end, with a pattern of acetylation [ADDA_n_−_3_] [55].

The above described CDAs are well characterized in terms of their deacetylation mode of action on COS and the structure of their deacetylated products. A number of other fungal CDAs (Table 1, and below) have also been assayed on COS substrates but, to the best of our knowledge, the deacetylation pattern of the products has not been reported.

*Saccharomyces cerevisiae* (*Sc*CDA1 and 2). The *S. cerevisiae* ascospore walls are well ordered structures with two outer layers that confer spore resistance, one made of 95% chitosan and the outermost proteinaceous layer rich in dityrosine [44]. Two CDAs are expressed exclusively during sporulation and are required for spore wall rigidity [38]. Both CDAs have been cloned and expressed in yeast as glycosylated proteins active on glycol-chitin [38,56], and in *E. coli* [57] as soluble proteins with deacetylase activity on glycol-chitin, chitosan (DA 50%) and COS. More detailed characterization of *Sc*CDA2 expressed in *E. coli* revealed that at least two GlcNAc residues are required for activity on COS, with maximum activity on DP6 [57]. When glycol chitin is used as substrate the optimum temperature for enzyme activity is 50 °C and the pH optimum is 8.0. It has also been shown that the *Sc*CDAs may act on nascent chitin chains in an in vitro assay system with chitin synthase [56].

*Mortierella* sp. (*Mo*CDA). Some *Mortierella* species live as saprotrophs in soil and other organic materials such as decaying plant leafs, fecal pellets or on the exoskeleton of arthropods, whereas other species are endophytes [111]. An extracellular CDA was identified [58] and purified from a *Mortierella* sp. as a highly glycosylated protein with maximum activity at pH 5.5–6 and 60 °C [112]. *Mo*CDA is active on soluble substrates as chitosans and glycol-chitin but with no detectable activity on β-chitin, colloidal chitin, and CM-chitin. It is active on COS with a DP ≥ 2, with higher activity with increasing DP of the substrate. With diacetylchitobiose, only monodeacetylation was observed. The structure of the deacetylated products from larger oligomers has not been reported.

*Absidia* sp. (*Acoe*CDA, *Acory*CDA). *Absidia* strains of *Zygomycetes* produce chitosan in their cell wall through the tandem action of chitin synthases and deacetylases. In *A. coerulea*, chitosan accounts for 10% of the vegetative cells and the DA reaches 95%. *AcoeCDA* was purified and proven to be active on glycol-chitin with a pH optimum of 5 at 50 °C. When the purified enzyme was incubated with a chitin synthase, it converted 90% of the nascent chitin from UDP-GlcNAc into chitosan. It deacetylates COS with more than two GlcNAc units, with increasing activity with longer substrates [43]. Similarly, *Absidia corymbifera* secretes a CDA active on glycol-chitin and chitosans with optimum pH and temperature of 6.5 and 55 °C, respectively, and active on COS with DP ≥ 2 [59].

*Flammulina velutipes* (*Fv*CDA). The basidiomycete *Flammulina velutipes* (called Enokitake in Japan) is commercially cultivated and fruited to produce foods with high nutritional value. A CDA that is expressed at the early stages of fruity body development was recombinantly expressed in *Pichia pastoris* [60]. *Fv*CDA, active on glycol-chitin and colloidal chitin, deacetylates COS from dimer to pentamer, with activity increasing with the DP of the substrate. The enzyme exhibits the maximum activity at 60 °C and pH 7.

*Penicilium oxilicum* (*Po*CDA). An extracellular CDA from *Penicilium oxilicum*, purified from culture supernatants, exhibits deacetylase activity on glycol-chitin at pH 9, a common value for extracellularly secreted CDAs as opposed to intracellular CDAs with typical pH optima in the range of 5 to 7. *Po*CDA is active on COS with activity increasing from DP2 to DP5 [61].

*Aspergillus flavus* (*Af*CDA). In the search for extracellularly secreted CDAs for industrial applications, optimization of solid substrate fermentation and submerged fermentation of *Aspergillus flavus* has been reported [62,113]. The *Af*CDA enzyme purified from the extracellular medium has optimal activity on glycol-chitin and colloidal chitin at pH 8 and 50 °C. When assayed with COS as substrates, *Af*CDA is active on DP4 but has no activity on shorter substrates [62].

*Scopulariopsis brevicaulis* (*Sb*CDA). *Scopulariopsis* spp. are common soil saprophytes. Few species have been associated with human diseases, including *S. brevicaulis*. They are dermatomycotic molds and mainly have been associated with onychomycosis [114,115]. *Sb*CDA is an extracellular enzyme that is active on chitin and chitosans. The purified native enzyme is also active on COS with at least two GlcNAc units, and the activity increases with the DP of the substrate. With DP6, optimum conditions for deacetylation are pH 7.5 and 55 °C [63].

*Rhizopus* sp. (*Rc*CDA, *Rs*CD*A*). *Rhizopus* species have been screened as CDA producers. A *R. circicans* CDA has been cloned and recombinantly expressed in *Pichia pastoris* [64]. *Rc*CDA has maximum activity on glycol-chitin at pH 5–6 and 37 °C. On COS, only activity on a DP6 substrate has been reported. A CDA from *Rhizopus stolonifer* (or *nigricans*) has also been isolated as an active enzyme on glycol-chitin but no activity on COS has been reported [64,116]. Fermentation conditions of other *Rhizopus* species as CDA producers are being studied for the bioconversion of chitin to chitosan [117].

Other chitosan producers have been identified and studied as a source of chitosans, with many reports on screening and fermentation optimization, but the corresponding chitin deacetylases have not been characterized yet. Some examples include *Gongronella butleri* [65,118], *Phycomyces blakesleeanus* [66,119], and *Schizophyllum commune* [67].

*Cryptococcus neoformans* (*Cn*CDA). *Cryptococcus neoformans* is a dimorphic basidiomycetous human fungal pathogen that causes cryptococcal meningoencephalitis, particularly in immunocompromised patients [37]. *C. neoformans* has substantial chitosan in its cell wall during vegetative growth that is necessary for virulence and persistence in the mammalian host [120,121]. Three CDAs are predicted to be GPI-anchored to the cell wall, suggesting that they transverse the plasma membrane or attach to the cell wall to deacetylate the chitin generated by a chitin synthase as it is extruded through the plasma membrane [37]. The GPI-anchor of *Cn*CDA2 has proven to be required for membrane association but dispensable for cell wall association [122]. Activity of *C. neoformans* CDAs on COS substrates has not been reported. Interestingly, screening studies to identify cryptococcal antigens that stimulate an immune response on murine T cell hybridomas reactive with cryptococcal proteins, have shown that two of the CDAs are immunogenic [123,124].

### 3.3. Protozoan CDAs

*Entamoeba histolytica* (*Eh*CDA). *Entamoeba histolytica* is an anaerobic parasitic amoebozoan that predominantly infects humans and other primates causing amoebiasis [125]. The genome contains two putative CDAs, one of which has been cloned and recombinantly expressed in *Saccharomyces cerevisiae* [68]. *Eh*CDA deacetylates COS, being active on DP5 and DP6, but with no detected activity on DP4 [68].

### 3.4. Bacterial CDAs

The predominant CE4 deacetylases in bacteria are chitin oligosaccharide deacetylases (CODs), active on low molecular mass COS and essentially inactive on polymeric chitin and chitosans. These include rhizobial NodB deacetylases and CODs from marine bacteria. But bacterial CDAs other than CODs are being discovered from screening programs and data mining of sequenced genomes and metagenomes, as in the recent case of an *Arthobacter* CDA.

*Sinorhizobium meliloti* (NodB). Rhizobial NodB is part of the Nod operon involved in the biosynthesis of Nod factors, the morphogenic signal molecules produced by rhizobia, which initiate the development of root nodules in leguminous plants [126]. NodB is active on chitooligosaccharides from DP2 to DP5 with no differences in k_cat_, but K_M_ decreases with increasing DP [18,127,128,129]. Specifically, k_cat_/K_M_ is 5-fold higher for DP5 than for DP2 substrates. DP4 or DP5 substrates are the natural substrates depending on the Rhizobial strain. *Sm*NodB optimum activity between pH 7 and 8 at 30 °C [18]. NodB is highly specific deacetylating only the non-reducing end residue [DA_n_−_1_] although traces of a second deacetylation event have been observed upon long incubations [18,130,131].

*Vibrio* species (*Vc*CDA, *Vp*CDA, *Va*CDA). Chitin oligosaccharide deacetylases (COD) from the *Vibrionaceae* family are involved in the chitin degradation cascades occurring in sea water [132,133,134,135]. They have been identified in many *Vibrio* species, such as *V. algynolyticus* [73,136], *V. parahaemolyticus* [72,137], *V. cholera* [70], *V. harveyi* [138] and others. The *V. parahaemolyticus* and *Vibrio* sp. SN184 CDAs only deacetylate DP2 and DP3 substrates, whereas the *Vibrio cholera* chitin deacetylase (*Vc*CDA) has a broader specificity, accepting substrate from DP2 to DP6 [69,70]. *VcCDA* has a 10-fold higher activity on DP2 than on DP4 [69], and specifically deacetylates the penultimate residue from the non-reducing end, generating monodeacetylated products with the pattern [ADA_n_−_2_] [69,70,130].

*Shewanella* species (*Sw*COD, *Sb*COD). In addition to the *Vibrio* genus, CODs have been recently identified and characterized from the *Shewanella* genus, marine bacteria found in extreme aquatic habitats (low temperature and high pressure). *Shewanella* sp. CODs share high sequence identity (50–60%) with *Vibrio* CODs, and have essentially the same biochemical properties. The *S. woodyi* enzyme (*Sw*COD) contains two CBM12 chitin binding domains at the C-terminus, deacetylates the reducing end on diacetylchitobiose [AD], and the activity drastically decreases from DP2 to DP4 substrates, with no activity detected on a DP5 substrate [74]. The *S. baltica* enzyme (*Sb*COD) contains a single CBM12 at the C-terminus, it is active of diacetylchitobiose with the same deacetylation pattern [AD] but it is less active on a DP3 than on a DP4 substrate [75].

*Arthrobacter* sp. (*Ar*CE4). A bioinformatics search for monodomain and extracellular CDAs in annotated genomes and metagenomes identified *Ar*CE4 as a CDA from an *Arthrobacter* species [76], a Gram-positive bacteria known to grow on chitin and secrete chitinases [139,140,141]. *Ar*CE4 is active on chitosan (DA 64%), acetylxylan, and insoluble chitin. It also deacetylates COS substrates with DP ≥ 2. The activity increases with increasing DP, with higher activity against DP5 compared to DP6. As shown with the DP5 substrate, the enzyme follows a multiple-chain mechanism where different mono- and di-deacetylated products are obtained. Whereas the first deacetylation occurs at all three internal positions, di-deacetylation mainly takes place at the GlcNAc unit next to the reducing end and at either of the two other internal units (ADDAA and ADADA). The final products have a pattern of acetylation [D_n_−_1_A], where the reducing end unit is not deacetylated [76].

## 4. Structural Determinants of Activity and Specificity

Structural analysis of CE4 enzymes with solved 3D structure have been recently reviewed [142], comparing and highlighting the differences between the different subfamilies based on substrate preferences. Here we focus and summarize the current knowledge on the structure and specificity of CDAs as a subfamily of CE4 enzymes. The closer similarity and activity on the same substrates provides a framework to analyze the structural determinants responsible for the different modes of action that lead to different patterns of deacetylation in their products.

### 4.1. 3D Structures

Some CDAs are mono-domain proteins and some others have a multi-domain architecture composed of the CE4 catalytic domain (or NodB homology domain), and several other domains, such as carbohydrate binding modules (CBMs [143]) and domains with unknown function. The function of the CBMs is not clear and might be diverse depending on the biological role of each enzyme in its organism. In extracellular CDAs acting on the cell wall chitin, they may facilitate solubilization and access to the substrate (i.e., *Pa*CDA with two CBMs, where deletion of one or both confirmed their proposed function in supporting the enzymatic conversion of insoluble chitin [26]). In CDAs acting on low molecular weight COS, the CBMs may be involved in enzyme localization. This is the case of COD enzymes from marine bacteria (*Vibrio* and *Shewanella* species), where the small substrate does not span out of the active site, and the CBMs might bind to chitinous material in order to keep the COD activity close to the site where COS are generated by the action of chitinases.

**Figure 2:**
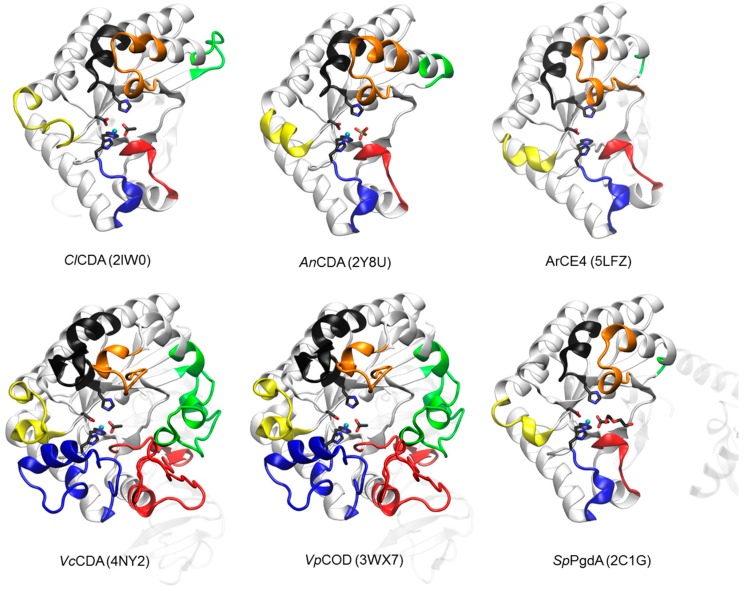
3D structures of CDAs determined by X-ray crystallography. Loops 1 to 6 colored as in Figure 3. The peptidoglycan deacetylase *Sp*PgdA is also included for comparison (see text). In parenthesis, PDB accession codes.

The first CE4 enzymes with 3D structure determined by X-ray crystallography were the peptidoglycan deacetylases *Bs*PdaA [144] and *Sp*PgdA [145], and the first CDA was that from *Colletotrichum lindemuthianum* (*Cl*CDA) [53]. Currently, only five CDAs in the CE4 family have known 3D structure (Figure 2). The CE4 catalytic domain is characterized by a distorted (β/α)_8_ barrel fold. The distorted barrel, which often lacks one of the αβ repeats of regular TIM barrels, creates a groove into which the extended polymer substrate binds [144,146,147]. Seven or eight parallel β-strands form the β-barrel surrounded by α-helices. In addition, a series of loops decorate the β-barrel and make up the majority of the carbohydrate binding pocket as discussed below.

### 4.2. The NodB Homology Domain and Conserved Active Site Motifs

**Figure 3:**
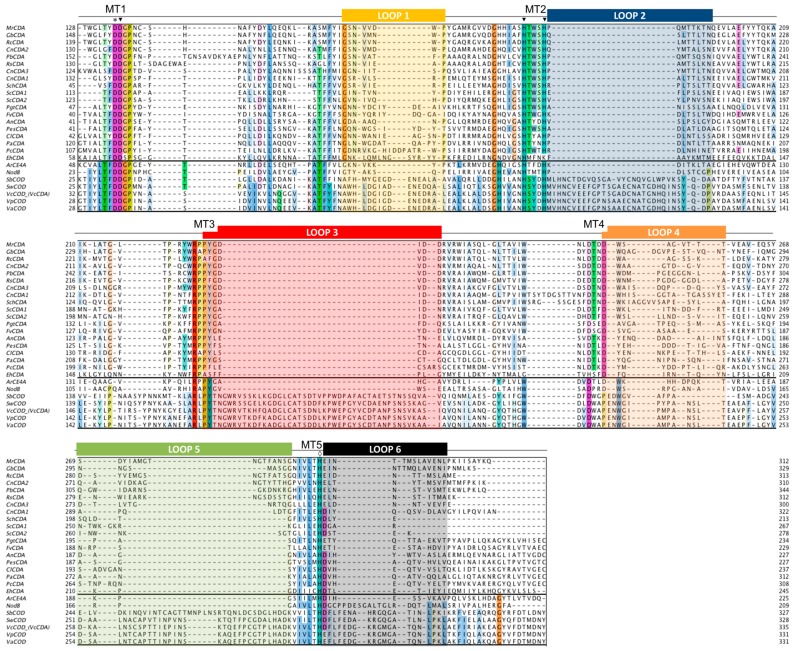
Multiple sequence alignment of the CDA enzymes listed in Table 1. Loops are highlighted with colored boxes according to [69]. Conserved catalytic motifs are labelled MT1-5. The ‘His-His-Asp’ metal binding triad (▼), catalytic base (*), and catalytic acid (◊) are labelled.

The multiple sequence alignment of the CE4 domain for the CDAs listed in Table 1 was guided by the structural superimposition of the available X-ray structures (Figure 2) and is presented in Figure 3. Compared to most of the CDA members, the *Vc*CDA enzyme has substantially longer insertions, and it was key to defining the loops that differentiate CDAs and shape the binding site cleft of these enzymes. Sequences of enzymes without structural date were incorporated into the alignment by means of Hidden Markov Model comparisons. As seen in Figure 3, the conserved motifs and non-conserved insertions are evenly distributed along the sequences of CDAs.

The conserved motifs are related to enzymatic activity (Motifs 1 to 5) and are typically located at the center of the active site structure. The non-conserved insertions correspond to both un-structured and structured loops of variable length, sequence, and geometry that surround the active site. These loops are numbered from Loop 1 to Loop 6 in the sequence alignment (Figure 3). As discussed in Section 4.5, they are key elements in determining the substrate specificity of different CDAs.

As members of the CE4 family, CDAs share the ≈150 aa-long NodB homology domain (CE4 domain). This region is defined by five conserved motifs that, according to the order they appear in the sequence, are named Motif 1 to Motif 5. These consensus motifs were first proposed in 2005 by sequence alignment of representative enzyme members of the CE4 family when the 3D structure of the peptidoglycan deacetylase *Sp*PgdA was solved [144]. Motif 1 (TFDD) is highly conserved in CDAs and contains the general base aspartate (first D) and the metal-binding aspartate (second D). Motif 2 (H(S/T)xxH) is a zinc-binding motif, where the two His residues bind the metal cation and the Ser or Thr residue forms a hydrogen bond with the second His, stabilizing the local conformation of the loop-shaped motif. These two His from Motif 2 plus the metal-binding Asp from Motif 1 are often designated the His-His-Asp metal-binding triad of CE4 enzymes. Motif 3 (RxPY) forms one of the sides of the active site groove and establishes stabilizing interactions with other active site residues. Motif 4 (DxxD(W/Y)) forms the other side of the active site groove, including a hydrophobic residue exposed to the solvent and a buried Asp. Motif 5 (I(V/I)LxHD) contains the catalytic general acid His residues and a Leu, which is part of a hydrophobic pocket that accommodates the acetate methyl group of the substrate.

### 4.3. Phylogeny of CE4 Chitin Deacetylases

**Figure 4:**
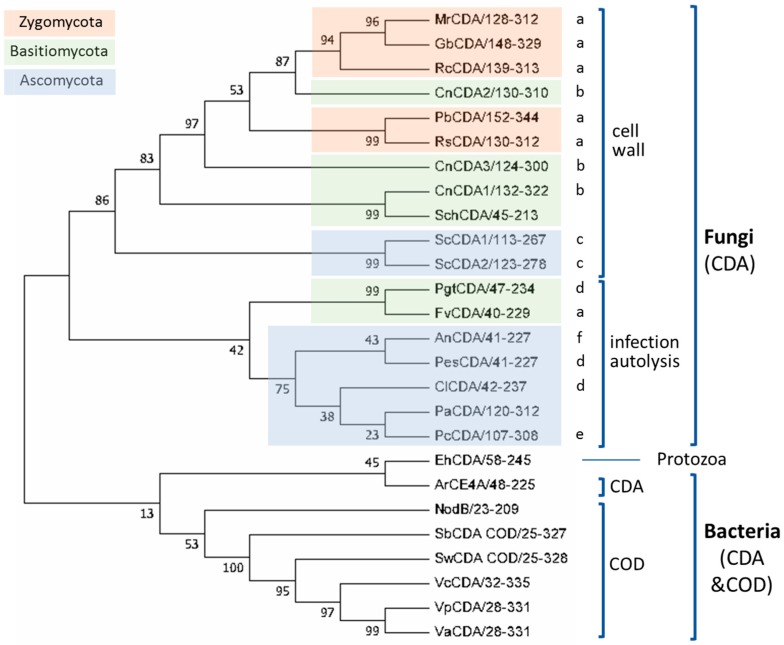
Phylogenetic analysis of CDAs from the multiple sequence alignment presented in Figure 3. A bootstrap analysis with 500 replicates was carried out on the trees inferred from the neighbor joining method. The consensus tree is shown with bootstrap values at each node of the tree. Biological functions: cell wall biosynthesis: (a) cell wall, (b) vegetative growth, (c) sporulation; host infection, (d) defense, (e) interaction/infection, (f) autolysis (see text).

Based on the above multiple sequence alignment, a clustering of the CE4 domain sequences of characterized CDAs based on phylogenetic analysis is presented in Figure 4. This is a reduced phylogenic analysis limited to the CDAs with reported activity on COS as listed in Table 1. Fungal and bacterial CDAs are clearly segregated in two clades, with a protist CDA (*Eh*CDA) located between both groups.

Fungal enzymes from organisms belonging to different phyla (*Zygomycota*, *Basidiomycota*, and *Ascomycota*) are distributed throughout the fungal clade. Within the clade, CDAs appear grouped in two clusters related with their biological function. The first cluster contains orthologous CDAs of different phyla known to have a role in cell wall chitosan biosynthesis at different stages of the fungal cell cycle, such as *Mr*CDA during cell wall formation [42], *Cn*CDAs during vegetative growth [37], or *Sc*CDAs during sporulation [38,44]. Although there is no experimental proof of their biological function, the CDAs from *Gonglonella*, *Phycomyces*, as well as those from *Rhizopus* are likely to be also involved in cell wall formation due to their location in the same cluster of the phylogenetic tree and their taxonomic classification (mucorales inside the *Zygomycota* phylum). Regarding the cellular location of the enzymes in this cluster, most of them are secreted to the periplasm or are GPI-anchored to the cell wall, where they are coupled with chitin synthases for chitosan biosynthesis. The second cluster is mainly composed of extracellular CDAs that participate in host infection, either as a defense mechanism to prevent the elicitation of host defense mechanisms (*Pgt*CDA, *Pes*CDA), or involved in the interaction with the host or as a virulent factor (*Cl*CDA, *Pc*CDA). Extracellular CDAs involved in cell autolysis (*An*CDA) also fall in this group.

Most of the bacterial enzymes included in the alignment are chitin oligosaccharide deacetylases (COD) and are more distantly related to the fungal CDAs. These form a different clade in the phylogenetic tree (Figure 4). The enzymes from marine bacteria (*Vibrio* and *Shewanella* species) are clustered together with high sequence similarity and have similar biological functions and biochemical properties. NodB has a more distant relationship with the other CODs.

### 4.4. Catalytic Mechanism

**Figure 5:**
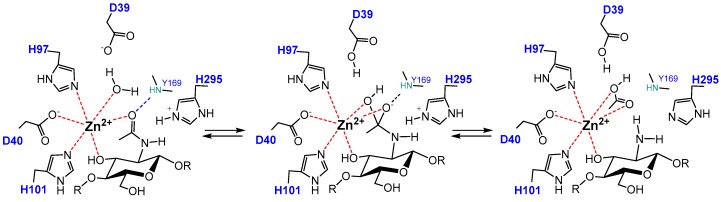
Metal-assisted general acid/base mechanism proposed for CE4 deacetylases. Scheme based on the 3D structure of the enzyme·substrate complex VcCDA_D39S_·DP2 [61]. D39 is the general base and His295 is the general acid.

CDA enzymes operate by metal-assisted acid/base catalysis. The general mechanism was first proposed for the peptidoglycan GlcNAC deacetylase *Sp*PgdA when solving its X-ray structure [144] and short after supported by the 3D structures of the acetylxylan esterases *Sl*AxeA and *Ct*AxeA [148]. The catalytic machinery involves the conserved active site motifs containing the metal-binding triad and the general acid and base residues. Only the structures of four different CDA have been solved up to date (*Colletotrichum*, *Aspergillus*, *Vibrio*, and *Arthrobacter* CDAs, Table 1), all consistent with the proposed metal-assisted mechanism. *Vc*CDA was the first CE4 enzyme for which the 3D structure of enzyme-substrate complexes were solved by X-ray crystallography [69]. The structure of complexes of an inactive mutant (at the general base Asp residue) with diacetylchitobiose (DP2) and triacetylchitotriose (DP3) in productive binding for catalysis showed that a sugar hydroxyl group of the substrate also participates in metal coordination. Specifically (Figure 5), the Zn^2+^ cation is coordinated by the imidazole nitrogens of His97 and His101, the carboxylate group of Asp40, and the O7 atom of the *N*-acetyl group and O3 hydroxyl of the GlcNAc ring. The distorted octahedral coordination is completed by a water molecule. Upon activation, this water molecule is proposed to be the nucleophile responsible for removal of the *N*-acetyl group. Just recently, a second structure of an enzyme-substrate complex has been reported for the *Arthrobacter* sp. CDA (*Ar*CE4) [76]. The diacetylchitobiose ligand bound into the active site also shows the same type of interactions with the conserved active site residues.

The proposed mechanism of CDAs and related CE4 enzymes is shown in Figure 5. In the first step, metal coordination polarizes the carbonyl amide of the substrate which reacts with the nucleophilic water molecule activated by the general base (Asp), leading to a tetrahedral oxyanion intermediate. Next, protonation of the nitrogen group of the intermediate by the general acid (His) facilitates C-N bond breaking with release of acetate and the generation of a free amine in product. Kinetic evidence for an oxyanion tetrahedral intermediate and significant charge development at the first transition state was provided by Hammett linear free energy correlations using the *Cl*CDA enzyme with α-haloacetamido substrate analogues [53]. In most of the enzymes, the catalytic acid and base residues are part of two conserved “charge relay” side chain pairs that may contribute to modulate the p*K*_a_ of the catalytic residues [53,144]: the catalytic base (Asp) is tethered by a conserved Arg from MT3 (RxxPY) and the catalytic acid (His) is tethered by a conserved Asp from MT4, DxxD(W/Y).

### 4.5. Determinants of Substrate Specificity

**Figure 6:**
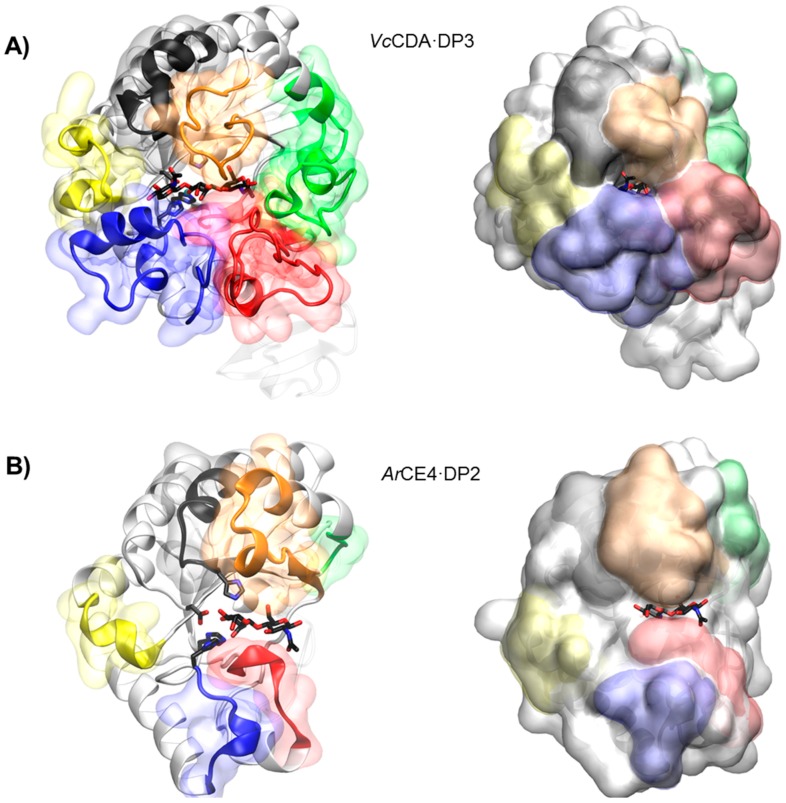
3D structures of enzyme·substrate complex. (**A**) *Vc*CDA with DP3 substrate and (**B**) *Ar*CE with DP2 substrate. Loops 1 to 6 are colored as in Figure 3.

The series of crystal structures of the *Vibrio cholerae* chitin oligosaccharide deacetylase (*Vc*CDA or *Vc*COD) reported in 2014 [69] were the first 3D structures of a CE4 enzyme in complex with substrates. These data provided a first insight into structure-function relationships for this family of enzymes and highlighted the role of the loops that shape the binding site cleft in substrate binding. Recently by the end of 2017, the 3D structure of an *Arthrobacter* sp. CDA (ArCE4) in complex with substrate has also been reported [76]. Albeit sharing the same molecular function, both enzymes represent two different scenarios regarding the binding site topology and, hence, substrate specificity. *Vc*CDA has a rather closed binding cleft and is highly specific for monodeacetylation of COS, whereas *Ar*CE4 has a more open binding cleft and is able to fully deacetylate their COS substrates (Figure 6). A comparative structural analysis of both enzyme structures has been recently reviewed [142].

#### 4.5.1. *Vc*CDA. Long Loops and High Specificity

Currently available structures of *Vc*CDA include the unliganded form of the enzyme and the binary complexes with *N*-acetylglucosamine (DP1), diacetylchitobiose (DP2), and triacetylchitotriose (DP3). These structures revealed two significant observations: a series of non-conserved loops (labeled Loop 1 to 6 in Figure 3 and Figure 6) that shape the binding cleft, and the dynamics of the loops that assemble the active site for catalysis [69].

In all structures, the substrate is confined in a small binding cleft that is shaped by a series of long loops surrounding the active site (see Figure 6A for the *Vc*CDA·DP3 complex). Given the topology of *Vc*CDA protein surface, the binding of longer COS is prevented because these loops cap both the reducing and non-reducing ends of the substrate. Indeed, the catalytic efficiency of *Vc*CDA drops substantially on oligomers longer than DP2 [69] and attempts to solve the structure of *Vc*CDA bound to substrates longer than DP3 in a catalytically competent mode have been unsuccessful. Another consequence of this constricting topology is the high specificity of *Vc*CDA to exclusively deacetylate the penultimate residue from the non-reducing end of the substrates. There is no room for the ligand to slide along the binding cleft, thus it can only accommodate one GlcNAc unit of the oligomeric chain at the catalytic site where deacetylation takes place. The binding of substrates induces a conformational change of Loop 4 from an open conformation in the unliganded enzyme to a closed conformation in the enzyme·DP2 complex or a semi-closed conformation in the enzyme·DP3 complex. It is triggered by a staking interaction between a Trp residue located in apical site of Loop 4 and the GlcNAc unit at the catalytic center, locking the substrate in the active site in the proper orientation for catalysis.

#### 4.5.2. *Ar*CE4. Short Loops and Broad Specificity

In contrast to *Vc*CDA, the crystal structure of *Ar*CE4 in complex with diacetylchitobiose [76] reveals a flatter protein surface with the substrate bound to a more open binding cleft (Figure 6B). Even though the enzyme was co-crystalized with tetraacetylchitotetraose (DP4), only two GlcNAc units are observed in the structure. This indicates a weak binding of part of the COS substrate on this flat topology of the protein surface. The catalytic center in both enzymes is in the same position with respect to the protein core, being the main difference, the size and shape of the loops surrounding the active site. Since the binding cleft is more open, the enzyme can accommodate longer COS. Indeed, the enzymatic activity of *Ar*CE4 increases as the length of the chitin oligomer chain increases. The lack of protein caps at either the reducing and non-reducing ends of the substrate can also explain the multiple-chain mechanism proposed for this enzyme. Deacetylation takes place at all GlcNAc units of the substrate (except the reducing end) because it can freely bind to *Ar*CE4 in different binding modes exposing different GlcNAc units of the oligomeric chain at the catalytic site.

#### 4.5.3. The Subsite Capping Model

The diversity of deacetylation patterns exhibited by chitin deacetylases and related CE4 enzymes can be attributed to the differential accessibility of the linear chitin oligosaccharide chain to the separate subsites along the substrate binding cleft of their structures. Considering all CE4 enzymes with reported activity on polymeric chitin or COS, these can be classified into two groups. One group is represented by general chitin deacetylases (CDA), and a second group is formed by chitin oligosaccharide deacetylases (COD). The two structures of the enzymes-substrate complexes described above are reference models for the protein surface topologies and substrate binding mechanisms of these two groups: CDAs (*Ar*CE4) and CODs (*Vc*CDA). These two structures provide a unified view of the determinants of substrate specificity in chitin deacetylases in terms of the “subsite capping model” proposed in [69]. According to this model, substrate accessibility is affected by the length, shape, and dynamics of a series of loops surrounding the active site of CE4 enzymes. These loops are numbered from 1 to 6 and their location in the sequences and structures of CDAs and CODs is highlighted in Figure 2 and Figure 3.

The group of CDAs bears short loops, and their structures exhibit a flat and open binding cleft. The substrate binding mechanism in this group of enzymes may be similar to that described for the reference structure of *Ar*CE4. According to the model, the substrate may be able to slide along the binding cleft or to bind in different modes resulting in processive or multiple-chain attack mechanisms of deacetylation. This can already be anticipated for CDA enzymes of known structure (Figure 2) because the flat protein surface is already evident, but also for CDA enzymes of unknown structure given the similar sequence lengths of the loops evidenced in the alignment (Figure 3). This could be an explanation of why CDA enzymes in general are not specific for the deacetylation at a single *N*-acetylglucosamine unit. However, the patterns of deacetylation differ among the different CDAs. The surface charge distribution along the binding cleft and other structural features yet to be disclosed may also participate in defining the mode of action and deacetylation pattern by each particular enzyme.

On the contrary, the group of COD enzymes bears longer loops and their structures have narrower binding pockets and buried active sites. According to the subsite capping model, the substrate is constrained to bind in very specific binding modes resulting in single-site deacetylations. This is the case for the reference structure of *Vc*CDA in complex with substrates, but it can also be anticipated for other COD enzymes for which the 3D structure is still unknown. For instance, Loop 6 in *Rm*NodB is longer than in other CDAs. This loop is located on the non-reducing end site of the binding cleft and may cap the accessibility of the substrate after subsite 0 (the catalytic site) thus defining the deacetylation specificity for the non-reducing end of the substrate. Likewise, the *Shewanella* CODs have a Loop 6 with the same length than the *Vibrio* CODs, but shorter than NodB, and both exhibit the same mono-deacetylation specificity for the penultimate GlcNAc residue from the reducing end of the substrate.

For most CDAs, the reducing end of the substrate is not deacetylated, or it is the least reactive GlcNAc unit. As seen in the *Ar*CE4·DP2 complex 3D structure [64], binding to the +1 subsite seems to be dominated by the stacking interaction of the GlcNAc unit of the substrate with a Trp in Motif 4 at the beginning of Loop 4. This aromatic residue is highly conserved (MT4, DxxD(W/Y), Figure 3). CDA enzymes having this aromatic residue prefer a sugar bound in the +1 subsite; they do not deacetylate the reducing end of their substrates, as it is the case for *Ar*CE4, *Pes*CDA [89] and *Pc*CDA [95], or the reducing end is the slowest position to be deacetylated, as shown for *Cl*CDA [49] and *An*CDA [12]. On the contrary, *Pgt*CDA, which deacetylates the reducing end GlcNAc unit of all substrates from DP4 to DP6, lacks the +1 aromatic residue [89]. Different is the case of *Vibrio* and *Sewanella* CODs that have the equivalent aromatic residue in a slightly different position after a two-amino acid insertion in the MT4 motif, and it is located farther in Loop 4 (Figure 3). In the *Vc*CDA enzyme this loop moves from an open to a closed conformation upon substrate binding, and the same is expected for the other closely related CODs that have the same Loop 4 size. As a consequence of the induced fit, the Trp residue now establishes a stacking interaction with the GlcNAc unit in subsite 0. DP2 is the preferred substrate for this group of COD enzymes, and it is deacetylated at the reducing end [62]. 

Gaining further structural information of protein-ligand complexes of CDA and COD enzymes, and other CE4 in general, will contribute further to decipher the structural and sequential determinants of substrate specificity in this family of enzymes. This will pave the way to the rational design or discovery of novel CDA with controlled specificities on the deacetylation of oligomeric and polymeric chitin for the biotechnological production of chitosans and paCOS with defined patterns of acetylation.

## 5. Application of Chitin Deacetylases

### 5.1. Targets for Antifungals

Fungal infections have an enormous impact on human health. Fungi are generally opportunistic pathogens affecting immunocompromised individuals including those with AIDS, receiving immunosuppressive drugs or undergoing cancer treatments. The cell wall is a meaningful target for antifungal therapies. Current major classes of antifungal drugs target cell membrane ergosterol biosynthesis (azoles), ergosterol function by disrupting membrane integrity (polyenes), or 1,3-β-glucan synthase preventing the formation of the cell-wall structural polysaccharide 1,3-β-glucan (echinocandins) [149]. New targets to overcome the emerging drug resistance by pathogenic fungi are becoming critical to treat life-threatening fungal infections. Other promising targets are the so called cell wall proteins (CWP) which mediate important cellular processes, including adhesion, invasion, biofilm formation and flocculation [122]. In fungal chitosan producers, chitin deacetylases are a class of CWP and potential targets for drug design. *Cryptococcus neoformans*, one of the most deadly pathogens, requires chitosan for virulence. Lack of chitosan in the cell wall has detrimental consequences in fungal growth and results in the complete loss of sporulation [120,121]. Thus, CDAs represent a promising target for anticryptococcal therapeutics [37,120], but no CDA inhibitors have been reported yet.

In pathogenic plants, major strategies to prevent fungal pathogenesis are related to the inhibition of fungal chitinases, which are required for chitin remodeling in the cell wall [150,151,152]. Different types of chitinase inhibitors have been reported, including potent natural inhibitors such as allosamidin [153] and the cyclic pentapeptides argifin and argadin [154]. However, inhibition must be selective so as not to interfere with the plant chitinases involved in triggering the plant defense mechanisms. Another potential and promising strategy is the inhibition of extracellularly secreted fungal CDAs since they constitute a defense mechanism to evade the plant immune system, as discussed in Section 3.2. As in the case of human fungal pathogens, no inhibitors have been yet reported against CDAs from plant pathogenic fungi.

### 5.2. Biocatalysts for the Enzymatic Production of Chitosans and paCOS

Chitosans can be found in a large number of applications in such distant areas as agriculture, cosmetics, water treatment, medicine and the food industry [155,156,157,158,159]. In addition to chitosan polymers, their oligomers (paCOS) have also proven to have relevant potential applications in agriculture and pharmaceutical industries [160]. The physicochemical and biological properties of chitosans and paCOS have been shown to be strongly dependent on their degree of polymerization and their degree of acetylation [161,162]. Many of the identified CDAs arose from screening programs addressed to find efficient biocatalysts to overcome the current industrial chitosan production by highly concentrated alkali treatment of chitin. Some examples include CDAs from *Mortiriella* sp. [58], *Rhizopus* sp. [117], or *Gongronella* sp. [118].

Chemical methods for the production of COS and paCOS are based on chemical depolymerization of chitosan [163,164], total synthesis of chitosan oligomers [165,166], partial chemical deacetylation of fully acetylated COS, or chemical re-*N*-acetylation of glucosamine oligomers based in two-step procedures or one-pot synthesis [167,168,169]. The drawbacks of chemical strategies are the unwanted side reactions and the randomness of the chemical reactions. Current efforts are addressed to develop enzymatic routes for COS and paCOS production with defined DP, DA, and PA.

**Figure 7:**
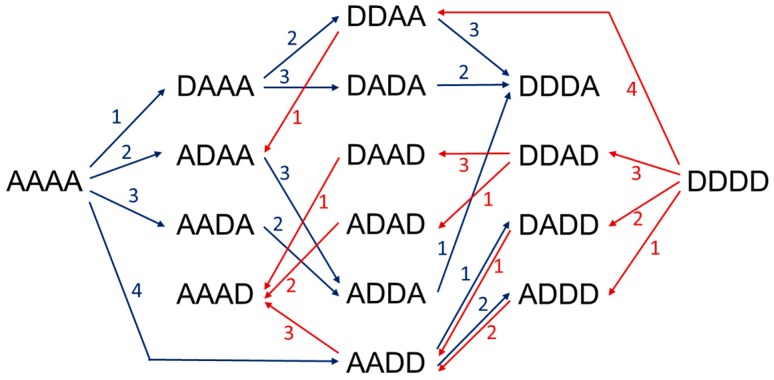
Production routes of all possible chitin and chitosan tetramers using 4 different CDAs to specifically deacetylate or *N*-acetylate paCOS. A: GlcNAc, D: GlcNH_2_. Blue arrows, deacetylation reactions, red arrows, *N*-acetylation reactions in the presence of excess acetate.

Enzymatic approaches include depolymerization of chitin or chitosan polymers using hydrolytic enzymes, chitinases and chitosanases, enzymatic polymerization by transglycosidation using transglycosylating hydrolases, and enzymatic de-acetylation and re-acetylation of chitin oligomers using chitin deacetylases, strategies recently reviewed in [170]. Based on the current knowledge on the specificity of a number of fungal and bacterial CDAs, recent reports have combined enzymes with different specificities to have access to a large family of paCOS with defined structures. The first proof of concept was to show that two specific CODs, NodB and VcCDA, each accept the monodeacetylated product from the other, leading to specific di-deacetylation, and that both enzymes can work in a one-pot process [130]. Recently, the use of different recombinant CDAs from bacterial and fungal origin to produce all of fourteen possible partially acetylated chitosan tetramers combining different enzymatic deacetylations and enzymatic *N*-acetylations has been reported [171] (Figure 7).

The production of paCOS using in vivo strategies is an alternative to increase the scalability of the process. The first example towards a more general cell factory approach for the in vivo synthesis of paCOS was based on NodB deacetylase. By in vivo studies with *Escherichia coli* expressing different combinations of the nodABCS genes of *Azorhizobium caulinodans*, Nod factor intermediates were identified, as well as the sequence of the biosynthetic steps [172]. The nod gene cluster encodes a series of enzymes, which include the NodC chitin oligosaccharide synthase that produces fully acetylated chitin oligomers, the NodB chitin oligosaccharide deacetylase that deacetylates the non-reducing end unit, the NodA *N*-acyl transferase that transfer a fatty acid chain to the free amine group, and the NodS *N*-methyl transferase. Further transformations by other nod proteins elaborate the final Nod signaling factors [173]. In a first cell factory approach, high density cells of *E. coli* expressing nodC or nodBC genes produced in high yield (up to 2.5 g/L) penta-*N*-acetyl-chitopentaose and its deacetylated derivative tetra-*N*-acetyl-chitopentaose, which were easily purified by charcoal adsorption and ion-exchange chromatography [174]. The strategy was further extended to the production of sulfated and *O*-acetylated derivatives of these two compounds by coexpressing nodC or nodBC with nodH and/or nodL that encode chitooligosaccharide sulfotransferase and chitooligosaccharide *O*-acetyltransferase, respectively [175]. Other Nod analogues have also been generated with further modifications [176,177,178]. The cell factory approach, currently limited to one deacelylation pattern based on the use of NodB, is a promising technology to be developed by incorporating the diversity of CDAs with different deacetylation patterns in order to access a large family of paCOS and derivatives.

## 6. Conclusions

We have here summarized the current knowledge on substrate specificity of fungal and bacterial chitin deacetylases, their modes of action, and their use as biocatalysts for the production of chitosans and chitosan oligosaccharides with defined pattern of acetylation. By combining multiple sequence alignments and 3D structures of enzyme·substrate complexes of representative enzymes, a unified view of the determinants of substrate specificity is proposed in terms of the “subsite capping model.” According to this model, substrate accessibility is affected by the length, shape, and dynamics of a series of loops surrounding the active site of CE4 enzymes. The group of CDAs active on polymeric substrates and COS bear short loops, and their structures exhibit a flat and open binding cleft. The substrate may be able to slide along the binding cleft or to bind in different modes, resulting in processive or multiple-chain attack mechanisms of deacetylation. Other structural features not yet disclosed, such as the charge distribution along the binding cleft may also participate in defining the mode of action and deacetylation pattern by each particular enzyme. The group of COD enzymes active on low molecular mass COS bear longer loops and their structures have narrower binding pockets and buried active sites. The substrate is constrained to bind in very specific binding modes resulting in single-site deacetylations.

But a deeper knowledge on substrate specificity requires further structural information of protein-ligand complexes of CDA and COD enzymes in order to decipher the structural and sequential determinants of substrate specificity in this family of enzymes aimed at the rational design or discovery of novel CDAs with controlled specificities on the deacetylation of oligomeric and polymeric chitin for biotechnological applications. 

Although CDAs have been proposed as targets for antifungal drugs, no specific inhibitors have been yet reported. This is an open field that deserves attention not only for drug design but also to probe the signaling function of CDAs and CODs through their specific deacetylation of COS substrates.

Applications of CDAs and CODs as biocatalyst are currently being developed as a novel methodology to produce partially acetylated COS with tailored patterns of acetylation. Since not all patterns for COS of different sizes are yet available, enzyme discovery and protein engineering offer new opportunities for the biotechnological production of chitosans and paCOS with defined patterns of acetylation.

## Figures and Tables

**Table 1:** CDAs and CODs with characterized activity on COS.

Enzyme ^1^	Organism	ID ^2^	PDB ^3^ [Ref.]	COS Substrates ^4^	Ref. ^5^ COS	Metal ^6^	PA ^7^ (on A_n_)
*Mr*CDA	*Mucor rouxii*	P50325		≥DP3	[31]	Zn^2+^	D_n_
*Cl*CDA	*Colletotrichum lindemuthianum*	Q6DWK3	2IW0 [53]	DP6 > DP5 > DP4 > DP3 > DP2	[32]	Co^2+^, Zn^2+^	D_n_
*An*CDA	*Aspergillus nidulans*	Q5AQQ0	2Y8U [25]	DP2 > DP3 > DP4 > DP5	[25]	Co^2+^	D_n_
*Pa*CDA	*Podospora anserine*	XP_001912680.1		≥DP2	[26]	Zn^2+^	D_n_
*Pgt*CDA	*Puccinia graminis*	XP_003323413.1		DP6 > DP5 > DP4	[54]	n.r. ^7^	AAD_n−2_
*Pes*CDA	*Pestolotiopsis* sp.	APH81274.1		DP6-DP5-DP4	[49]	n.r.	AAD_n−3_A
*Pc*CDA	*Pochonia chlamydosporia*			DP5 > DP4	[55]	n.r.	ADDA_n−3_
*Sc*CDA1	*Saccharomyces cerevisiae*	Q06702		DP4, DP6	[56]	n.r.	n.r.
*Sc*CDA2	*Saccharomyces cerevisiae*	Q06703		DP6 > DP5 > DP4 > DP3 > DP2	[57]	Co^2+^	n.r.
*Mo*CDA *	*Mortierella* sp.			DP7 > DP6 > DP5 > DP4 > DP3 > DP2	[58]	(Co^2+^)	n.r.
*Acoe*CDA *	*Absidia coerulea*			DP5 > DP4 > DP3	[43]	n.r.	n.r.
*Acor*CDA *	*Absidia corymbifera*			DP7 > DP6 > DP5 > DP4 > DP3 > DP2	[59]	(Co^2+^, Ca^2+^, Mg^2+^)	n.r.
*Fv*CDA	*Flammulina velutipes*	BAE92728.1		DP5 > DP4 > DP3 > DP2	[60]	(Co^2+^, Ca^2+^, Zn^2+^)	n.r.
*Po*CDA *	*Penicillium oxilicum*			DP5 >> DP3 > DP2	[61]	(Co^2+^ Cu^2+^)	n.r.
*Af*CDA *	*Aspergillus flavus*			DP4	[62]	(Zn^2+^, Mn^2+^)	n.r.
*Sb*CDA *	*Scopulariopsis brevicaulis*			DP6 > DP5 > DP4 > DP3 > DP2	[63]	n.r.	n.r.
*Rc*CDA	*Rhizopus circinans*	A7UMZ0		DP6	[64]	(Mn^2+^, Mg^2+^)	n.r.
*Rs*CDA	*Rhizopus stolonifer (nigricans)*	Q32XH4		n.r.	[64]		
*Gb*CDA	*Gongronella butleri*	Q8J2N6		n.r.	[65]		
*Pb*CDA	*Phycomyces blakesleeanus*	Q9P4U2		n.r.	[66]		
*Sch*CDA	*Schizophyllum commune*	Q9P453		n.r.	[67]		
*Cn*CDA1, 2, 3	*Cryptococcus neoformans*	Q5KFG8, Q5KIC2, P0CP76		n.r.	[37]		
*Eh*CDA	*Entamoeba histolytica*	XP_656356.1		DP5, DP6	[68]	n.r.	n.r.
NodB	*Sinorhizobium meliloti*	P02963		DP5 > DP2 (DP4, DP3)	[18]	Mn^2+^ Mg^2+^	DA_n−1_
*Vc*COD (*Vc*CDA)	*Vibrio cholera*	Q9KSH6	4NY2 [69]	DP2 > DP3 > DP4 > DP5 > DP6	[70]	Zn^2+^	ADA_n−2_
*Vp*COD	*Vibrio parahaemolyticus*	Q9KSH6	3WX7 [71]	DP2 > DP3	[72]	Zn^2+^	n.r.
*Va*COD	*Vibrio alginolyticus*	Q9KSH6		DP2	[73]	Zn^2+^	AD
*Sw*COD	*Shewanella woodyi*	ACA84860.1		DP2 > DP3 > DP4	[74]	n.r.	AD; [ADA_n−2_]
*Sb*COD	*Shewanella baltica*	ABN60929.1		DP2 > DP4 > DP3	[75]	n.r.	AD; [ADA_n−2_]
*Ar*CE4A	*Arthrobacter* sp.	A0A2C8C1T7	5LFZ [76]	DP5 > DP6 ≈ DP4 > DP3 >> DP2	[76]	Ni^2+ 8^	D_n−1_A

^1^ Characterized recombinant enzymes, except those with an asterisk (*) that have been characterized from the native organism and are not included in the sequence alignment, Figure 4. ^2^ Uniprot or GenBank accession code, ^3^ PDB accession code and publication of the 3D structure. ^4^ Activity on chitooligosaccharides (COS) as a function of the degree of polymerization (DP). ^5^ Selected publication on substrate specificity. ^5^ Native metal cation or, in parenthesis, metals that enhanced the enzyme activity when added in the reaction buffer. ^6^ Pattern of acetylation (PA): structure of the main final deacetylated product (A: GlcNAc; D: GlcNH_2_). Other patterns of acetylation with specific substrates are given in the text. ^7^ n.r.: not reported. ^8^ Native metal unknown, Ni^2+^ probably from purification/crystallization.

## Abbreviations

AXE	Acetylxylan esterase
A_n_	(GlcNAc)_n_
CDA	Chitin deacetylase
COS	Chitooligosaccharides
D_n_	(GlcNH_2_)_n_
DA	Degree of acetylation
DP	Degree of polymerization
GlcNAc	*N*-acetylglucosamine
PA	Pattern of acetylation
paCOS	Partially acetylated chiton oligosaccharides
PDB	Protein data bank
